# The Efficacy of Chinese Medicinal Herbs towards Grape Phylloxera (*Daktulosphaira vitifoliae* Fitch) (Hemiptera, Phylloxeridae)

**DOI:** 10.1371/journal.pone.0128038

**Published:** 2015-07-17

**Authors:** Yongqiang Liu, Zhongyue Wang, Junping Su, Weiwei Liu, Khalid Hussain Dhiloo, Yuyuan Guo

**Affiliations:** State Key Laboratory for Biology of Plant Diseases and Insect Pests, Institute of Plant Protection, Chinese Academy of Agricultural Sciences, Beijing, 100193, P. R. China; Universita degli Studi di Siena, ITALY

## Abstract

Bio-insecticidal effects of seven Chinese medicinal herbs on mortality, fecundity, developmental periods and life table parameters of the grape phylloxera were investigated. In an excised root bioassay experiment aqueous extracts from seven Chinese medicinal herbs increased grape phylloxera first instar mortality (26.00–38.50%) compared to other instars. The intrinsic rate of increase (r_m_), finite rate of increase (λ), fecundity rate and net reproductive rate (R_0_) were significantly reduced by *A*. *bidentata*, *A*. *tataricus*, *O*. *basilicum*, *P*. *frutescens* and *N*. *cataria*. In a glasshouse pot trial, eggs, nymphs, adults and total population were significantly reduced before population establishment compared to those after its population established, by *A*. *bidentata*, *A*. *tataricus* and *O*. *basilicum*. Overall, *A*. *bidentata*, *A*. *tataricus *and *O*. *basilicum* can be used to suppress all life-stages of grape phylloxera.

## Introduction

Grape phylloxera, *Daktulosphaira vitifoliae* (Fitch) (Hemiptera, Phylloxeridae), is an aphid-like pest, native to eastern North America but nowadays it is widely regarded as the most destructive insect pest of commercial grapevines *Vitis* worldwide [1]. Root-feeding stages are the most economically damaging and the subterranean habitat makes control difficult [2]. In the 19^th^ century, European vineyards were ravaged by these pests. Grape phylloxera was first reported as a grape pest in France and spread across continental Europe and finally around the world [3]. Since the 1920s, some studies on biology and control of grape phylloxera have been conducted worldwide.

Since the early historical outbreaks, grape phylloxera has been managed effectively using resistant rootstocks [2]. However, this strategy is facing a challenge as resistance of rootstocks to grape phylloxera that is being questioned as a result of genotype-genotype interactions between the host and pest. For example, host-associated grape phylloxera genetic clones with a preference for different *Vitis* genotypes has been observed in endemic Californian, German, Austrian, Australia and Hungarian grape phylloxera strains under laboratory conditions as well as under field conditions Californian [2, 4]. Moreover, the emergence of grape phylloxera biotype B caused a breakdown in the resistance of the widely planted rootstock AXR#1 (*V*. *vinifera* ‘Aramon’×*V*. *rupestris*) and cost the viticulture industry between US$ 1 and 6 billion [5–6].

There has been limited use of alternative chemical, biological and cultural control options for over nine decades, due to restrictions to high mammal’s toxicity insecticides and the insects’ subterranean habitat, no fully effective alternative control strategy has been developed for root-galling grape phylloxera in commercial vineyards. [2,4,7–9]. It is therefore necessary to explore more effective control options.

One potential option is to use intercropping plants with insecticidal activity. Intercropping is the simultaneous production of two or more crops in the same field [10]. Historically it has significantly contributed to agriculture and plays an important role in sustainable agriculture. Nowadays, intercropping based on the insecticidal characteristics of plant itself or its secondary metabolism is being used commonly to overcome agricultural pest problems globally [11–12]. In the case of phylloxera control it would be necessary to identify and utilize high efficacy plants which can be intercropped with grapevines.

More than 1500 plants species have been reported possessing insecticidal value[13], however, intercropped with grapevines required the plants have shade tolerance in order to complete the entire growth period under the grape arbor. Therefore, the present study was conducted to compare the effects of aqueous root extracts to grape phylloxera from seven Chinese medicinal herbs which have insecticidal activity [13–20], shade tolerance and can be artificially cultivated.

## Materials and Methods

### Insects and plants

With the authorization of Huaihua Agriculture Bureau, Hunan Province, grape phylloxera were collected from phylloxera-infested vineyards near Shuangxi town, Huaihua city, Hunan Province, China (27°14′N, 109°51′E). Post collection, phylloxera were maintained on excised grapevine roots petri dishes (12-cm diameter) in controlled environment incubators set of 26 ± 1°C, 80 ± 5% RH and a photoperiod of 0:24 h (L:D). Fresh excised roots (3–7 mm in diameter and 5 cm in length) of *Vitis labruscana* Kyoho were used to maintain and allow for the development of phylloxera population. One end of each root was wrapped in wet cotton to prevent desiccation. The maintenance method was followed according to de Benedictis and Granett [21].

The grape cultivar used in the study was ‘Kyoho’ (*Vitis*. *vinifera* L. × *V*. *Labrusca* L. cv. Kyoho), that is one of the main grape cultivar grown commercially in Hunan Province, China.

Seven Chinese medicinal herbs from four families (Table 1) with known insecticidal properties were tested in the present study [22]. Seeds or young plants of the selected herbs were purchased from Anguo Chinese medicinal herbs market (Hebei, China) and then planted in the experimental field plots near Shuangxi town, Huaihua city, Hunan Province, China (27°14′N, 109°51′E).

**Table 1:** Selected Chinese medicinal herbs with known insecticidal activity.

Chinese medicinal herbs	Family	Common name	Pharmaceutical name	Target insects
*Achyranthes bidentata* Blume	Amaranthaceae	Two tooth Achyranthes Root	Radix Achyranthis Bidentatae	*Chironomus tentans* [13]
*Aster tataricus* L. F.	Compositae	Tatarian Aster Root	Radix Asteris	*Pieris rapae* [38]
*Ocimum basilicum L. var. pilosum* (Willd.) Benth.	Labiatae	Fineleaf Schizonepeta Herb	Radix Schizonepetae	*Aedes aegypti* [39]
*Perilla frutescens* (L.) Britton	Labiatae	Perilla Leaf	Folium Perillae Argutae	*Sitophilus zeamais* [15]
*Nepeta cataria* L.	Labiatae	Catnip	Herba Schizonepetae	*Aedes albopoctus, Culex pipien pallens* [14]
*Mentha haplocalyx* Briq.	Labiatae	Mint	Radix Scurellariae	Ants, Mosquitoes, Wasps, Hornets and Cockroaches [42]
*Cassia obtusifolia* L.	Leguminosae	Semen Cassiae	Semen Cassiae Obtusifoliae	*Pieris rapae* [41]

### Grapevine root-dip bioassay

Fresh roots were obtained from mature Chinese medicinal herbs in the experimental field plots between 16–20^th^ of August, 2012. After washing with sterilized water, the roots were dried at 50°C for 24 h and ground using a Chinese medicine grinder (Deqing Baiji Electrical Appliance Co., Ltd., China) and then filtered to a fine powder using a 40 mesh stainless steel sieve. For extraction, 10 g of powdered roots were dissolved in 1:20 (w/v) of distilled water and vibration extracted for 12 h at 25°C. The mixture was stored at 25°C for 12 h and then filtered by centrifugation (10 min, 3000 g). The filtered root extracts were stored in the refrigerator at 4°C for the further studies [23].

A root dipping method, adopted from a leaf-dip bioassay method [24] was used to determine the effect of Chinese medicinal herbs aqueous root extraction against grape phylloxera. Grapevine roots were obtained from the vineyard in the experimental field plots. Roots of (3–7 mm in diameter and 5 cm in length) were washed with distilled water. Each root piece was immersed in aqueous herb root extracts for 5 min and then dried on tissue paper in a fume hood for 1.5 h. After drying, the roots were placed in pairs on filter paper discs in glass petri dishes (12 cm diam.). One end of each root was wrapped in wet cotton to prevent desiccation. All of the petri dishes were maintained in controlled environment incubators as mentioned above (26 ± 1°C, 80 ± 5% RH) and a photoperiod of 0:24 h (L:D). Grape phylloxera eggs (approximately 6 h old) were selected from the laboratory colony and placed on each grape root in petri dishes which were sealed as to prevent insect escaping or cross contamination. Seven herb root extract treatments and one control treatment were used and in each treatment about 200 eggs were exposed, i.e. 50 eggs were considered as one replicate and four replicates per treatment. Bioassay plates were checked after every 24 h, and hatched and unhatched eggs were counted. The eggs were considered as unviable if no hatching occurred during the experiment. The mortality and survival of nymphs, adult survival and number of eggs laid were recorded on daily basis. The nymph instar was estimated based on its size [25]. The experiments continued until the death of each individual.

Life table parameters including intrinsic rate of increase (r_m_), finite rate of increase (λ), net reproductive rate (R_0_), mean generation time (T) and population doubling time (DT) were calculated.

### Potted grapevine bioassay

One year-old grapevine seedlings were potted in 6L pots filled with culture substrates composed of soil, fowl manure and sand at a 7: 1: 1 ratio. Sixty days after transplanting, the potted grapevines were used in two different experiments.

Potted experiment 1: A total of 200 grape phylloxera eggs were first placed onto the excised grapevine roots (3–7 mm in diameter and 5 cm in length), then were placed near the grapevine roots in the pots and Simultaneously before the phylloxera population established, twenty plants of the medicinal herbs were transplanted into each potted grapevine. The numbers of eggs, nymphs and adults per plant were checked for 120 days after inoculation.

Potted experiment 2: A total of 200 phylloxera eggs were first placed onto the excised grapevine roots (3–7 mm in diameter and 5 cm in length), then were placed near the grapevine roots in the pots and 60 days later after its population established, twenty plants of the medicinal herbs were transplanted into each potted grapevine. The numbers of eggs, nymphs and adults per plant were counted for 60 days after inoculation.

In both experiments, each treatment was replicated 3 times, with 5 potted grapevines for each replication. The potted grapevine monocultures were used as control.

### Data analysis

The following life table parameters were calculated for grape phylloxera for all Chinese medicinal herbs: the net reproductive rate, (*R*_*0*_) = ∑*l*_*x*_ *m*_*x*_; the intrinsic rate of increase (*r*_*m*_), which was calculated by iteratively solving the equation ∑*l*_*x*_ *m*_*x*_ *e* ^*-rmx*^ = 1; the mean generation time, (*T*) = ln*R*_*o*_/*r*_*m*_; doubling time, (DT) = ln (2)/r_m_; and the finite rate of increase, (*λ*) = *e* ^*rm*^. In the equations, *l*_*x*_ is the age-specific survival rate, which is the probability to survive to a particular age x, and *m*_*x*_ is the age-specific fecundity, which is calculated as the number of live females per female for age x [26]. All data were analyzed by SPSS 13.0 (SPSS Inc., Chicago). Egg hatching rate, nymphs (1^st^, 1^st^ -2^nd^, 1^st^ -3^rd^, 1^st^ -4^th^ and 1^st^, 2^nd^, 3^rd^, 4^th^ instar) mortality, the data on development rate of each stage of the grape phylloxera at different Chinese medicinal herbs, life table parameters and control efficiency of 3 Chinese medicinal herbs to total population and different development stages of grape phylloxera in potted test were analyzed by one-way ANOVA. If the ANOVA indicated a significant difference, Tukey's HSD (honestly significant difference) test was followed to separate the means. Differences of the control efficiency in potted test before and after its population established were analyzed by *t*-tests. All the proportion data were square root arcsine transformed before processed.

## Results

### Nymph mortality

**Fig 1:**
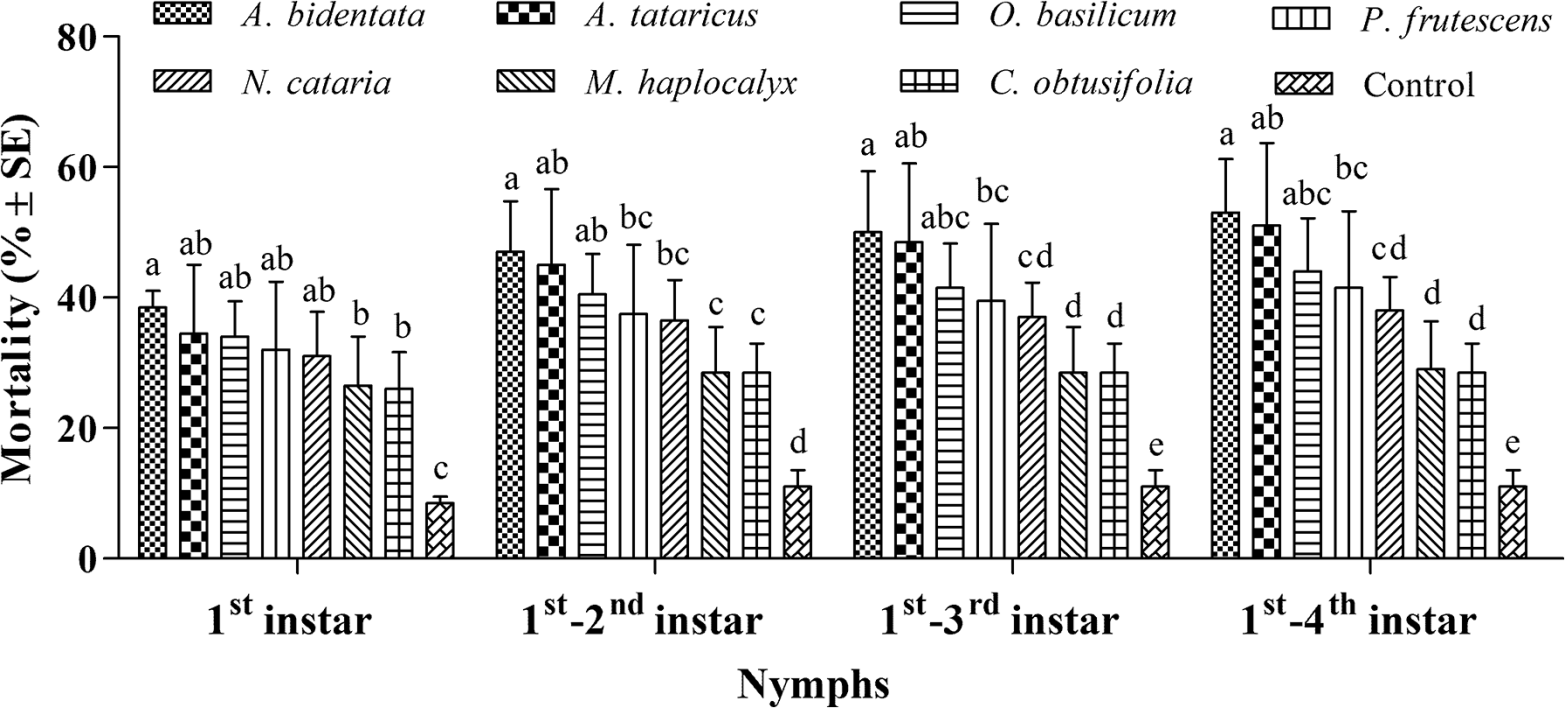
Mean mortality (%±SE) of grape phylloxera eggs, on grapevine root treated with aqueous root extract of Chinese medicinal herbs. Within each diagram and instars, means followed by the same letters are not significantly different (Tukey’s HSD test, P>0.05).

In the excised root bioassay no significant hatching differences (*F* = 0.87, *d*.*f*. = 7, 24, *P* = 0.55) were observed for all treatments including control, with 86.50 ± 5.00, 87.50 ± 4.43, 89.50 ± 4.43, 91.00 ± 4.76, 91.00 ± 3.46, 92.00 ± 2.83, 92.00 ± 5.16, and 92.00 ± 4.32% for *A*. *bidentata*, *A*. *tataricus*, *O*. *basilicum*, *P*. *frutescens*, *N*. *cataria*, *M*. *haplocalyx*, *C*. *obtusifolia* extracts and control, respectively. There were significantly higher mortality between treatments and control for 1^st^ instar (*F* = 8.78, *d*.*f*. = 7, 24, *P* < 0.001), 1^st^ -2^nd^ instar (*F* = 10.93, *d*.*f*. = 7, 24, *P* < 0.001), 1^st^ -3^rd^ instar (*F* = 11.47, *d*.*f*. = 7, 24, *P* < 0.001) and 1^st^ -4^th^ instar nymphs (*F* = 12.94, *d*.*f*. = 7, 24, *P* < 0.001) mortality. All seven Chinese medicinal herbs extracts showed higher activity to 1^st^ instar nymphs than other nymphal stages (*A*. *bidentata*: *F* = 73.28, *d*.*f*. = 3, 12, *P* < 0.001; *A*. *tataricus*: *F* = 20.42, *d*.*f*. = 3, 12, *P* < 0.001; *O*. *basilicum*: *F* = 64.85, *d*.*f*. = 3, 12, *P* < 0.001; *P*. *frutescens*: *F* = 20.26, *d*.*f*. = 3, 12, *P* < 0.001; *N*. *cataria*: *F* = 67.36, *d*.*f*. = 3, 12, *P* < 0.001; *M*. *haplocalyx*: *F* = 43.63, *d*.*f*. = 3, 12, *P* < 0.001; *C*. *obtusifolia*: *F* = 71.65, *d*.*f*. = 3, 12, *P* < 0.001). All seven Chinese medicine herbs extracts showed effective results on nymphs of grape phylloxera and *A*. *bidentate*, *A*. *tataricus* and *O*. *basilicum* extracts have higher effects to them, whereas, there were no significantly differences among *A*. *bidentate*, *A*. *tataricus* and *O*. *basilicum* (Fig 1).

### Effect of seven Chinese medicinal herbs on developmental period

Table 2 shows the effect of treatments on life history parameters of grape phylloxera extracts on life history statistics of grape phylloxera.

**Table 2:** Life history parameters (mean ± SE) of grape phylloxera treated by aqueous root extract of seven Chinese medicinal herbs.

Treatments	Egg incubation (days)	1^st^ instar (days)	2^nd^ instar (days)	3^rd^ instar (days)	4^th^ instar (days)	Adults (days)	Total life span (days)	Fecundity (eggs)
*A. bidentata*	6.29 ± 0.17 a	18.73 ± 0.48 d	6.54 ± 0.37 d	1.62 ± 0.09 b	1.16 ± 0.08 b	20.38 ± 0.45 d	55.12 ± 0.79 a	125.56 ± 7.66 e
*A. tataricus*	6.22 ± 0.10 a	17.94 ± 0.49 cd	6.29 ± 0.20 d	1.75 ± 0.22 b	1.18 ± 0.03 b	21.29 ± 0.91 d	53.86 ± 0.75 a	138.49 ± 4.17 de
*O. basilicum*	6.20 ± 0.09 a	16.67 ± 0.44 bc	5.41 ± 0.12 c	1.35 ± 0.07 ab	0.94 ± 0.06 b	23.05 ± 0.32 d	53.81 ± 0.61 a	151.61 ± 6.29 cde
*P. frutescens*	6.19 ± 0.05 a	15.24 ± 0.32 ab	5.18 ± 0.17 bc	1.06 ± 0.04 a	0.94 ± 0.01 b	24.78 ± 0.38 cd	53.66 ± 0.55 a	160.10 ± 8.27 cde
*N. cataria*	6.11 ± 0.06 a	15.11 ± 0.38 ab	4.78 ± 0.17 bc	1.06 ± 0.05 a	0.90 ± 0.06 b	25.41 ± 0.71 cd	53.74 ± 0.93 a	173.24 ± 6.25 bcd
*M. haplocalyx*	6.03 ± 0.02 a	14.90 ± 0.17 a	4.54 ± 0.13 ab	0.97 ± 0.07 a	0.89 ± 0.03 b	25.66 ± 0.62 bc	52.89 ± 0.79 a	178.24 ± 11.25 abc
*C. obtusifolia*	6.01 ± 0.00 a	14.68 ± 0.24 a	4.33 ± 0.10 ab	0.93 ± 0.04 a	0.88 ± 0.03 a	26.23 ± 0.25 ab	53.01 ± 0.46 a	188.13 ± 3.45 ab
Control	6.00 ± 0.03 a	14.53 ± 0.27 a	3.73 ± 0.03 a	0.93 ± 0.04 a	0.84 ± 0.02 a	26.35 ± 0.35 a	52.53 ± 0.61 a	201.24 ± 2.88 a

Seven Chinese medicinal herbs extracts didn’t have any egg developmental period (*F* = 1.84, *d*.*f*. = 7, 24, *P* = 0.13) and total life span (*F* = 1.29, *d*.*f*. = 7, 24, *P* = 0.3). First instar developmental period was significantly prolonged by *A*. *bidentata A*. *tataricus* and *O*. *basilicum* extracts (*F* = 19.36, *d*.*f*. = 7, 24, *P* < 0.001). Second (*F* = 27.30, *d*.*f*. = 7, 24, *P* < 0.001), third (*F* = 11.21, *d*.*f*. = 7, 24, *P* = 0.3) and fourth instars (*F* = 9.70, *d*.*f*. = 7, 24, *P* < 0.001) developmental period was also significantly delayed by *A*. *bidentata* and *A*. *tataricus* extracts compared to the control second and fourth instar developmental period was significantly delayed by *O*. *basilicum* and by *O*. *basilicum*, *P*. *frutescens*, *N*. *cataria and M*. *haplocalyx* respectively compared to the control. Adult developmental periods were significantly reduced by *A*. *bidentata*, *A*. *tataricus*, *O*. *basilicum*, *P*. *frutescens*, *N*. *cataria* and *M*. *haplocalyx* extracts as compared to the control (*F* = 18.03, *d*.*f*. = 7, 24, *P* < 0.001).

In addition, phylloxera fecundity was significantly reduced by *A*. *bidentata*, *A*. *tataricus*, *O*. *basilicum*, *P*. *frutescens* and *N*. *cataria* extracts compared to the control (*F* = 14.07, *d*.*f*. = 7, 24, *P* < 0.001).

### Life table parameters

The data on life table parameters viz., ‘r_m_’, ‘λ’, ‘R_0_’, ‘T’ and ‘DT’ of grape phylloxera treated by aqueous root extract of seven chinese medicinal herbs are presented in Table 3.

**Table 3:** Life table parameters (mean ± SE) of grape phylloxera treated by aqueous root extract of Chinese medicinal herbs.

Treatments	Intrinsic rate of increase (r_m_)	Finite rate of increase (λ)	Net reproductive rate (R_0_)	Mean generation time (T)	Population doubling time (DT)
*A. bidentata*	0.085 ± 0.005 e	1.089 ± 0.005 e	42.335 ± 10.919 c	43.523 ± 1.187 d	8.127 ± 0.457 d
*A. tataricus*	0.097 ± 0.015 de	1.102 ± 0.017 de	65.121 ± 46.008 bc	41.590 ± 1.298 cd	7.267 ± 1.013 cd
*O. basilicum*	0.106 ± 0.001 cd	1.112 ± 0.002 cd	69.915 ± 12.218 bc	39.782 ± 1.299 bc	6.514 ± 0.089 bc
*P. frutescens*	0.120 ± 0.009 bc	1.127 ± 0.010 bc	100.377 ± 29.529 bc	38.172 ± 0.473 ab	5.814 ± 0.438 ab
*N. cataria*	0.118 ± 0.006 bc	1.125 ± 0.007 bc	91.650 ± 12.910 b	38.281 ± 1.128 ab	5.889 ± 0.303 ab
*M. haplocalyx*	0.129 ± 0.005 ab	1.137 ± 0.005 ab	111.835 ± 20.270 ab	36.614 ± 0.779 a	5.400 ± 0.201 a
*C. obtusifolia*	0.132 ± 0.004 ab	1.142 ± 0.005 ab	118.220 ± 12.285 ab	36.021 ± 0.645 a	5.238 ± 0.177 a
Control	0.139 ± 0.005 a	1.149 ± 0.005 a	163.755 ± 16.951 a	36.616 ± 0.656 a	4.984 ± 0.161 a

The intrinsic rate of increase ‘r_m_’ was significantly decreased by *A*. *bidentata A*. *tataricus*, *O*. *basilicum*, *P*. *frutescens* and *N*. *cataria* extracts compared with the control (*F* = 25.69, *d*.*f*. = 7, 24, *P* < 0.001).

The finite rate of ‘λ’ increase was reduced by *A*. *bidentata A*. *tataricus*, *O*. *basilicum*, *P*. *frutescens* and *N*. *cataria* extracts as compared to the control (*F* = 25.79, *d*.*f*. = 7, 24, *P* < 0.001). Net reproductive rate was significantly lower from *A*. *bidentata*, *A*. *tataricus*, *O*. *basilicum*, *P*. *frutescens* and *N*. *cataria* extracts, compared with the control (*F* = 10.58, *d*.*f*. = 7, 24, *P* < 0.001).

The mean generation time ‘T’ increased significantly when treated with *A*. *bidentata A*. *tataricus* and *O*. *basilicum* extracts, compared with the control (*F* = 28.98, *d*.*f*. = 7, 24, *P* < 0.001). Population doubling time ‘DT’ was significantly longer when treated with *A*. *bidentata*, *A*. *tataricus* and *O*. *basilicum* extracts, compared with the control.

### Effect of selected Chinese medicinal herbs on grape phylloxera on potted grapevines

Results in Table 4 show that before the grape phylloxera population established, the total population was significantly reduced by *A*. *bidentata*, *A*. *tataricus* and *O*. *basilicum* compared with the control (*F* = 161.75, *d*.*f*. = 3, 8, *P* < 0.001). Similarly reduced egg (*F* = 191.38, *d*.*f*. = 3, 8, *P* < 0.001), nymph (*F* = 24.43, *d*.*f*. = 3, 8, *P* < 0.001) and adult (*F* = 290.37, *d*.*f*. = 3, 8, *P* < 0.001).

**Table 4:** The effects of Chinese medicinal herbs to grape phylloxera in potted grapevine bioassay before its population established.

Treatments	No. of grape phylloxera per plant			
	Eggs	Nymphs	Adults	Total population
*A. bidentata*	19.87 ± 1.92 c	20.27 ± 1.14 b	8.80 ± 1.80 c	48.93 ± 3.21 c
*A. tataricus*	25.54 ± 5.50 bc	26.79 ± 8.80 b	11.28 ± 1.86 bc	63.61 ± 11.32 bc
*O. basilicum*	32.24 ± 4.26 b	30.94 ± 9.08 b	16.50 ± 4.48 b	79.69 ± 15.32 b
Control	82.69 ± 0.30 a	68.44 ± 8.34 a	63.22 ± 0.71 a	214.36 ± 7.57 a

Post-phylloxera establishment (Table 5), the total population was significantly decreased by *A*. *bidentata*, *A*. *tataricus* and *O*. *basilicum* compared with the control (*F* = 9.11, *d*.*f*. = 3, 8, *P* = 0.006). The number of eggs also significantly lower when treated with *A*. *bidentata*, *A*. *tataricus* and *O*. *basilicum*, respectively (*F* = 17.15, *d*.*f*. = 3, 8, *P* < 0.001). There was no significant difference found in nymph abundance (*F* = 2.24, *d*.*f*. = 3, 8, *P* = 0.16). However, adult abundance was significantly decreased by *A*. *bidentata* and *A*. *tataricus* treatments.

**Table 5:** The effects of Chinese medicinal herbs to grape phylloxera in potted grapevine bioassay after its population established.

Treatments	No. of grape phylloxera per plant			
	Eggs	Nymphs	Adults	Total population
*A. bidentata*	79.80±5.33 b	76.27 ± 5.71 a	36.33 ± 3.07 b	192.40 ± 13.87 b
*A. tataricus*	88.33 ± 4.10 b	79.13 ± 7.72 a	48.27 ± 4.39 b	215.73 ± 16.07 b
*O. basilicum*	92.87 ± 5.25 b	79.73 ± 4.16 a	51.80 ± 3.34 ab	224.40 ± 12.73 b
Control	127.00 ± 15.09 a	91.87 ± 12.10 a	73.07 ± 15.84 a	291.93 ± 42.36 a

**Fig 2:**
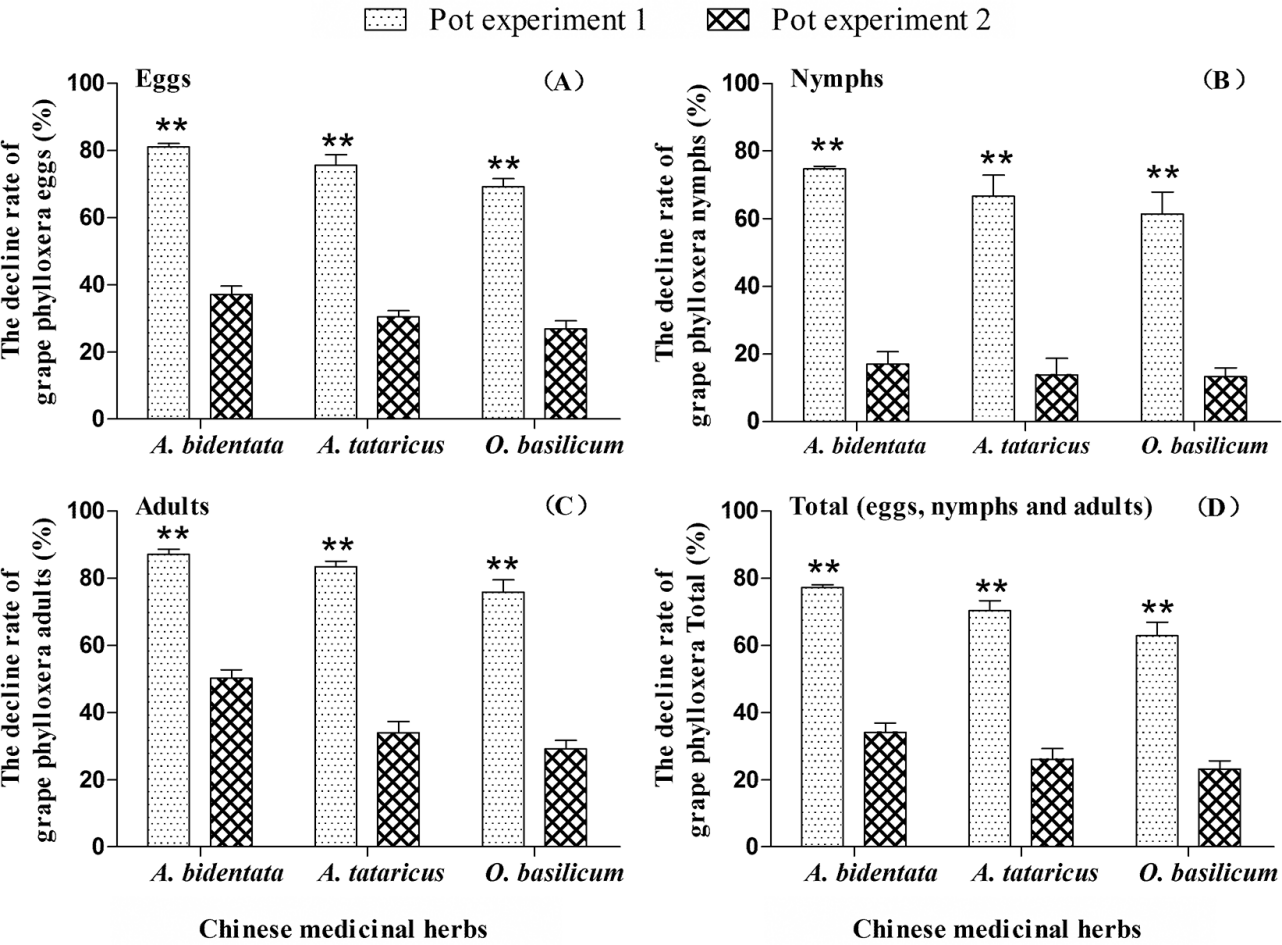
The decline rate of grape phylloxera eggs (A), nymphs (B), adults (C) and total population (eggs, nymphs and adults) (D) on potted grapevines before (pot experiment 1) and after (pot experiment 2) its population established. ** indicate a significant difference determined by t-test.

Potted test showed that the total population of grape phylloxera was comparatively decreased more before its population established by *A*. *bidentata* (77.17 ± 1.50%), *A*. *tataricus* (77.32 ± 5.28%) and *O*. *basilicum* (62.82 ± 7.15%) than after its population established by *A*. *bidentata* (34.09 ± 4.75%), *A*. *tataricus* (26.10 ± 5.50%) and *O*. *basilicum* (23.13 ± 4.36%) (*A*. *bidentata*: *t* = 14.65, *df* = 4, *P* < 0.001; *A*. *tataricus*: *t* = 9.32, *df* = 4, *P* = 0.001; *O*. *basilicum*: *t* = 7.89, *df* = 4, *P* = 0.001) (Fig 2D). Similar differences were observed in eggs (*A*. *bidentata*: *t* = 13.63, *df* = 4, *P* < 0.001; *A*. *tataricus*: *t* = 8.56, *df* = 4, *P* = 0.001; *O*. *basilicum*: *t* = 8.57, *df* = 4, *P* = 0.001)(Fig 2A), nymphs (*A*. *bidentata*: *t* = 11.45, *df* = 4, *P* < 0.001; *A*. *tataricus*: *t* = 4.83, *df* = 4, *P* = 0.008; *O*. *basilicum*: *t* = 5.29, *df* = 4, *P* = 0.006)(Fig 2B) and adults (*A*. *bidentata*: *t* = 11.78, *df* = 4, *P* < 0.001; *A*. *tataricus*: *t* = 11.85, *df* = 4, *P* < 0.001; *O*. *basilicum*: *t* = 8.53, *df* = 4, *P* = 0.001)(Fig 2C).

## Discussion

In this study, we tested the activity of seven Chinese medicinal herbs, using aqueous root extracts to grape phylloxera in the laboratory by excised root bioassay. We then evaluated the efficacy of three selected Chinese medicinal herbs against grape phylloxera in a potted grapevine test. In the excised root bioassay all seven Chinese medicinal herbs, *A*. *bidentata A*. *tataricus*, *O*. *basilicum*, *P*. *frutescens* and *N*. *cataria* showed significantly high effects towards grape phylloxera. In the potted grapevine bioassays, all of the selected three Chinese medicinal herbs (*A*. *bidentata A*. *tataricus* and *O*. *basilicum*) exhibited high control efficacy to grape phylloxera before its population established. Therefore, the selected three Chinese medicinal herbs have potential for the control of grape phylloxera.

The results revealed the insecticidal effects of seven Chinese medicinal herbs aqueous root extraction on grape phylloxera. The extracts increased nymph mortality, increased the nymph development time and decreased its fecundity. Similar observations have been reported using other plant extracts for several insects pests. Neem seed oil, *Eichlornia crassipes* (Ponteriaceae) and *Dysoxylum malabaricum* Bedd. (Meliaceae) extracts showed larvicidal activity against *Aphis glycines* (Glover), *Tribolium castaneum* (Herbst) and *Anopheles stephensi* Liston, respectively [27–29]. The developmental time of *Plutella xylostella* (L.) (Lepidoptera: Yponomeutidae), *Tribolium castaneum* (Herbst) and *Pieris rapae* (L.) (Lepidoptera: Pieridae) was significantly prolonged by neem, *Eichlornia crassipes* (Ponteriaceae) and *Rhododendron molle* (G. Don.) (Ericaceae) flower extracts, respectively [27, 30–31]. Jeyabalan et al. [32] also reported that *Pelargonium citrosa* (Vanleenii) (Geramiaceae) extracts prolonged the duration of larval instars of *Anopheles stephensi* (Liston) (Diptera: Culicidae). Fecundity of *Aphis glycines* (Homoptera, Aphididae) and *Tribolium confusum* (Jacqueline DuVal) (Coleoptera: Tenebrionidae) was significantly reduced by azadirachtin [29, 33]. Reduction in fecundity of *Sitophilus granaries* (L.) (Coleoptera: Curculionidae), *S*. *zeamais* (L.) and *Prostephanus truncatus* (Horn) (Coleoptera: Bostrychidae) was also observed when exposed to powdered leaves of *Chenopodium ambrosioides* (L.) (Chenopodiaceae) [34].

The intrinsic rate of increase (r_m_) is a measure of the ability of a population to increase exponentially in an unlimited environment. It provides an effective summary of an insect’s life history traits [35] and has also been recommended together with toxicity assessment to provide a more accurate estimate of population-level effect of toxic compounds [36–38]. In our study, r_m_ is positive and was significantly reduced by *A*. *bidentata A*. *tataricus*, *O*. *basilicum*, *P*. *frutescens* and *N*. *cataria* root extracts. This means that the grape phylloxera population exponential rate of increase when treated by Chinese medicinal herbs is lower than the control [39]. So these Chinese medicinal herbs root extracts significantly delayed the population increase of grape phylloxera.

Reproduction rate (R_0_) of grape phylloxera was significantly reduced by *A*. *bidentata A*. *tataricus*, *O*. *basilicum*, *P*. *frutescens* and *N*. *cataria* in the present study. This decline is regarded as a function of pesticide [40], therefore, *A*. *bidentata A*. *tataricus*, *O*. *basilicum*, *P*. *frutescens* and *N*. *cataria* demonstrated insecticidal properties to grape phylloxera. Doubling time of populations may show effects on increase in the time it takes for survivors to compensate for loss of individuals [41]. Doubling time of grape phylloxera was prolonged between 6.514–8.127 days by *A*. *bidentata A*. *tataricus* and *O*. *basilicum* extracts as compared to 4.984 days in control. So the population of grape phylloxera treated by three Chinese medicinal herbs needs more time to compensate for loss of individuals.

Under potted bioassay conditions, three Chinese medicinal herbs (*A*. *bidentata*, *A*. *tataricus* and *O*. *basilicum*) showed higher control efficiency towards grape phylloxera pre-population establishment than post-population establishment. This may be caused by treatments having a higher activity to 1^st^ instar nymphs than other instars (Fig 2). In terms of future management options this result indicates that the timing of application of Chinese medicinal herbs should focus on the period before its population established. Flooding of vineyards during the winter can decrease phylloxera populations [42]. Therefore, flooding during winter, combined with Chinese medicinal herbs intercropping in vineyards may potentially be used for grape phylloxera control. Further tests of the combination of these two methods, under field conditions need to be conducted.

Plant extracts with insecticidal activity have repellent, antifeedant and growth regulation effects upon insects, as they can affect insect physiology in diverse ways [13]. *A*. *bidentata*, *A*. *tataricus*, *O*. *basilicum* and *P*. *frutescens* extracts showed lethal effects to larvae of *Chironomus tentans*, *Pieris rapae*, *Aedes aegypti* and adults of *Sitophilus zeamais*, respectively. Meanwhile, *O*. *basilicum* and *P*. *frutescens* extracts have repellent effects to mosquitoes, *A*. *bidentate* and *A*. *tataricus* extracts have antifeedant effects to *Chironomus tentans* and *Pieris rapae* larvae, respectively [14, 16–18]. *M*. *haplocalyx* oil demonstrated repellency, adulticidal, larvicidal, growth and reproduction inhibition activity to various insects/pests [19]. Ren et al. [20] reported that trypsin inhibitor (COTI) from *C*. *obtusifolia* extracts had inhibitory effects on the development of *Pieris rapae*. Our results showed seven Chinese medicinal herbs extracts have insecticidal activities and growth regulation effects to grape phylloxera nymphs, but the mode of action of the plant extracts or their constituents, as insecticides is not clearly known. However, knowing the kinds of the secondary metabolites, their mechanisms of action are important for optimizing the application of the Chinese medicinal herbs, which requires a further research.

This study will provide valuable information on the potential use of intercropped Chinese medicinal herbs ccontaining secondary metabolites, which could effectively control grape phylloxera. The intercropping of plants with insecticidal activities and grapes should be enhanced greatly as a new strategy for sustainable management of grape phylloxera.
